# Cognitive and neurophysiological assessment of patients with minimal hepatic encephalopathy in Brazil

**DOI:** 10.1038/s41598-020-65307-3

**Published:** 2020-05-25

**Authors:** Daniel Simplicio Torres, Jefferson Abrantes, Carlos Eduardo Brandão-Mello

**Affiliations:** Federal University of the State of Rio de Janeiro (UNIRIO), Rio de Janeiro, RJ Brazil

**Keywords:** Neuroscience, Psychology, Biomarkers, Diseases, Gastroenterology, Medical research, Neurology, Signs and symptoms

## Abstract

Minimal hepatic encephalopathy is a syndrome caused by cirrhosis, with a broad spectrum of clinical manifestations. Its diagnosis is based on abnormal results of cognitive and neurophysiological tests, but there are no universally available criteria, especially in Brazil, where local testing standards are required. The objective of the present study was to compare the performance of the mini-mental state examination (MMSE), Rey’s auditory-verbal learning test (RAVLT), psychometric score of hepatic encephalopathy (PHES), topographic mapping of brain electrical activity (TMBEA) and long-latency auditory evoked potential (P300) in the detection of minimal hepatic encephalopathy in Brazil. From 224 patients with cirrhosis included in the global sample, 82.5% were excluded due to secondary causes responsible for cognitive or neurophysiological dysfunction. The final sample consisted of 29 cirrhotics, with predominance of A5 Child-Pugh classification, and 29 controls paired in critical variables such as age, educational level, gender, professional category, scores suggestive of mild depression, association with compensated type 2 diabetes mellitus and sociodemographic characteristics. Overall, performance on cognitive tests and TMBEA did not show a statistically significant difference. There was a marked difference in P300 latency adjusted for age, with patients with cirrhosis showing a mean of 385 ± 78 ms (median of 366.6 ms) and healthy volunteers exhibiting a mean of 346.2 ± 42.8 ms (median of 348.2 ms) (*p* < 0.01). These findings suggest that, in the earliest stages of cirrhosis, age-adjusted P300 latency was superior to cognitive assessment and TMBEA for detection of minimal hepatic encephalopathy.

## Introduction

Although, since ancient Babylonian times (1894 B.C. – 1595 B.C.), humanity has been aware about the effects of the liver on cognition^1^, until today little is understood about how the metabolic, cellular and immunological changes resulting from a dysfunction in such a distant organ affect the central nervous system. It is known that liver failure and portosystemic shunt that occur in cirrhosis are key components in the development of hepatic encephalopathy. This condition has a wide spectrum of neurological and psychiatric abnormalities, ranging from subclinical changes to coma^2^, but little is known about the neurophysiological processes that underlie them.

In this sense, one of the most difficult conditions to be studied in clinical practice is minimal hepatic encephalopathy, which affects 30 to 50% of patients with chronic liver disease^3^. Part of this is because it produces the most subtle neurological and psychiatric changes in the disease, which are difficult to analyse subjectively. At the beginning of the new millennium, significant efforts have been made trying to objectively detect, quantify and differentiate minimal hepatic encephalopathy from other conditions that affect cognition^2,3^. Hereupon, several psychometric and neurophysiological tests were described. However, their applicability has shown great variability according to sociodemographic differences of the studied populations.

Another factor that aggravates the pre-existing obstacles is that there are only few studies in literature that consider the possibility of overlapping minimal hepatic encephalopathy with other conditions that also produce neurophysiological and cognitive deficits^3^. In those where such confounding biases are taken into account, rates of up to 84% of the selected sample exhibited at least one concomitant condition that justified or worsened cognitive impairment^4^.

So far, there is no gold standard for diagnosing minimal hepatic encephalopathy^5^. Although, traditionally, it has been described as a pathology that affects the information processing speed, attention and motor coordination^6,7^, there are sufficient reasons to suspect that other cognitive functions may be altered, and that even minimal deficits may have great impact on quality of life, with reduced learning, ability to drive and increased caregiver burden^3^.

The Vienna consensus, developed in 2003 by the Organisation Mondiale de Gastroentérologie, considered cognitive tests, topographic mapping of brain electrical activity (TMBEA) and long-latency auditory evoked potential (P300) as valid resources for its detection^8^. In literature, there are few studies in which the three methods were used at the same time, and their results are conflicting^9,10^.

There is doubt about the usefulness of the psychometric hepatic encephalopathy score (PHES), mini-mental state examination (MMSE) and Rey’s auditory-verbal learning test (RAVLT) as screening instruments for minimal hepatic encephalopathy^11,12^. PHES is a battery of five tests that asses predominantly attention, psychomotor processing speed, and executive functions^13^, while MMSE provides relevant information on overall cognitive performance (including memory, attention, language, and visuospatial skills), and RAVLT better studies the mechanisms of memory coding, storage, and recall^14,15^.

Regarding PHES, there is great variability in the calculation of the score of one component of the battery, the line-tracing test (LTT). It consists in drawing a line inside a maze, in which the execution time (in seconds) and the number of errors (where the line touches the walls of the maze) are registered. For example, the German and Chinese versions calculate its score using the equation Z score = [time × (1 + number of errors/100)]^16,17^, while the Italian version multiplies the execution time by the number of errors^18^ and the Spanish and Portuguese versions add the execution time to the number of errors^19,20^.

In the face of so much controversy and the absence of a Brazilian consensus on the sensitivity, specificity and accuracy of the available diagnostic methods, aggravated by the lack of information on the influence of age, gender, educational level and sociodemographic profile on existing psychometric and neurophysiological tests, investigations are needed. The primary objective of the present study was to compare the performance of MMSE, RAVLT, PHES (German, Chinese, Italian, Spanish and Portuguese versions), TMBEA and P300 in the detection of minimal hepatic encephalopathy in paired samples in Brazil. The secondary objectives were to describe the cognitive functions and neurophysiological parameters that were the most compromised in the initial stages of cirrhosis, to compare the performance of cirrhotic patients with the performance of healthy controls, and to identify the standardized tests that detected these changes.

## Methods

Patients with cirrhosis aged 17 to 79 years were selected from the Hepatology Outpatient Clinic of the Gaffrée and Guinle University Hospital for a cross-sectional study with paired control group. These patients were selected according to one or more of the following inclusion criteria: percutaneous liver biopsy confirmatory of cirrhosis based on METAVIR classification;^21^ ultrasound of the liver with decreased parenchyma, heterogeneous echotexture, irregular contours and increased vascular tortuosity; transient hepatic elastography greater than or equal to 12.5 kPA;^22^ high-resolution digestive endoscopy showing esophageal varices or portal hypertensive gastropathy.

In patients with cirrhosis, the following were considered to be exclusion criteria: less than 2 years of schooling, a C Child-Pugh classification^23^, episodic or persistent liver encephalopathy in the previous 6 months, use of interferon in the previous 6 months, use of psychotropic medication, use of alcohol in the previous 6 months, use of illicit drugs in the previous 6 months, chronic obstructive pulmonary disease, neurological disease, psychiatric disease, neoplastic disease, thyroid disease, type 1 diabetes mellitus, decompensated type 2 diabetes mellitus, cardiac insufficiency, renal insufficiency, adrenal insufficiency, visual deficiency, folic acid deficiency, vitamin B12 deficiency, being seropositive for syphilis or HIV, infection within the previous 2 weeks, or digestive hemorrhage in the previous 2 weeks. Compensated type 2 diabetes mellitus was not an exclusion criterion.

The control group consisted of healthy individuals which were selected to match the patient group with respect to age (±3 years), gender, education (±1 year), sociodemographic characteristics, and proportion of patients with compensated type 2 diabetes mellitus. Subjects were interviewed to exclude those with visual deficiency, less than 2 years of schooling, use of interferon, use of psychotropic medication, use of illicit drugs in the previous 6 months, chronic obstructive pulmonary disease, neurological disease, psychiatric illness, neoplastic disease, thyroid disease, type 1 diabetes mellitus, decompensated type 2 diabetes mellitus, cardiac insufficiency, renal insufficiency, adrenal insufficiency, malnourishment, syphilis, HIV infection, viral hepatitis, auto-immune hepatitis, or a history of heavy drinking. Minimal and moderate drinking were not exclusion criteria. Both in patients and control group, the inclusion and exclusion criteria were similar to those of a study previously carried out in the same unit^4^. The control group was also paired with cirrhotics according to the proportion of white-collar (administrative or bureaucratic activities) and blue-collar (activities that require the use of physical force) professions.

Before starting the evaluation, patients and controls were interviewed to identify each group’s principal complaints. The questionnaire that was used addressed the following items: decreased attention; reduction in reasoning speed; difficulty performing tasks; difficulty in remembering things; decreased mental capacity as a whole; and other spontaneous neurological complaints.

Participants were asked to respond to the Beck Depression Inventory-II (BDI-II) survey. In Brazil, scores between 0 and 11 correspond to absent or minimal depression, 12 to 16 correspond to mild depression, and more than 16 correspond to moderate to severe depression^24^. Individuals receiving a score of more than 16 were excluded and sent for outpatient tracking. The control group was paired to the cirrhotic group so that there was no statistically significant difference in the proportion of patients with mild depression in BDI-II.

The remaining patients and controls completed the mini-mental state examination (MMSE), Rey’s auditory-verbal learning test (RAVLT) and psychometric hepatic encephalopathy score (PHES). PHES consists of five items: digit-symbol test (DST); line tracing test (LTT); serial dotting test (SDT); trail-making test A (TMT-A); and trail-making test B (TMT-B). Regarding LTT and its Z score, PHES was calculated according to German, Chinese, Italian, Spanish and Portuguese versions.

After cognitive evaluation, participants performed topographic mapping of brain electrical activity (TMBEA) and long-latency auditory evoked potential (P300). The equipment used in the study was a BNT-Plus electroencephalogram, which has 20 monopolar channels and an electrocardiogram channel. The record was captured by a MEDCAP cap (54 to 58 cm), which has plater electrodes arranged in bipolar longitudinal montage, which facilitates the preparation for the procedure. Impedances were kept below 5 kOhms, with 7 sensitivity, 0.3 Hz low frequency filter, 70 Hz high frequency filter, 60 Hz notch filter and 30 mm/s paper speed. Twenty periods of 2.56 seconds without sleep or drowsiness were selected for spectral analysis to exclude artifacts. The target variables were: mean dominant frequency (Hz), relative frequencies (%), absolute amplitudes (µV), and relative amplitudes (%). The theta/beta ratio, measured at cranial vertex (Cz) at rest, was recorded to assess the relative contribution of the theta and beta frequency bands.

The method used to evaluate P300 was the oddball paradigm, which consists of presenting a series of auditory stimuli, divided into frequent and rare. The hearing aid used to generate the stimuli was a 300 P sound stimulator, which is coupled to the BNT-Plus electroencephalogram. Phillips SHP 2500 headphones were used for listening. The record was captured by the same MEDCAP cap mounted on Fpz (ground), Fp1, Fp2, Fz, Cz, Pz and ear electrodes as reference. The exam was performed in four consecutive steps: trying to identify the stimuli presented; trying to identify the rare stimulus raising the hand; trying to count the rare stimuli; and trying to identify the rare stimulus raising the hand and counting at the same time.

In the present study, 192 frequent stimuli and 48 rare stimuli were applied in a pseudorandom pattern, with a 60 Hz notch filter. The responses generated by them allow distinguishing two P300 components: P3a and P3b. Latency was measured from the beginning of the auditory stimulus to the P300 peaks P3a and P3b, and the latency of the P3b component was adjusted according to age (since the latter may cause a physiological increase), using the equation Z score = {[value found − (250 + 1.4 x age)]/40}, which follows the standards advocated by the International Federation of Clinical Neurophysiology^25^. Amplitude, in turn, was measured from the N200 (N2) component nadir, up to the peak of P3b, being represented in microvolts (µV)^26^. The total time spent on cognitive and neurophysiological assessment was 2 hours.

Blood was collected from each patient with cirrhosis to assay levels of thyroid stimulating hormone (TSH), free thyroxine (free T4), cortisol, folic acid, vitamin B12, and to perform blood tests for syphilis (VDRL) and HIV. At this point of the research, the following were considered criteria for exclusion: TSH > 5 µU/ml, free T4 < 0.8 ng/dl, cortisol < 5 µg/dl, folic acid < 5 ng/ml, vitamin B12 < 250 pg/ml, and seropositivity for syphilis or HIV. Hemoglobin levels (Hb) and hematocrits (Hct) were also recorded to exclude cases of severe anemia (e.g., Hb < 7 g/dl or Hct < 21%). Again, these exclusion criteria were similar to those of a study previously carried out in the same unit^4^.

R (v. 3.3.1) was used to analyse the data. The Kolmogorov-Smirnov test rejected the hypothesis of normal distribution of the sample. To compare the performance of patients with cirrhosis to the control group, the non-parametric Mann-Whitney U test (for more than two categorical variables) and Fisher’s exact test (for two categorical variables) were applied. The chi-square test was used to compare proportions between the groups, such as prevalence of cognitive complaints, gender, professional category, compensated type 2 diabetes mellitus and scores suggestive of mild depression in BDI-II. The Kruskal-Wallis test was used to verify whether any characteristic of the patients under study (such as age, gender, educational level, professional category, severity of cirrhosis, etiology, compensated type 2 diabetes mellitus, mild depression or interferon use) influenced their performance. Descriptive levels with significance less than 5% (*p* < 0.05) were considered statistically significant.

### Ethical issues

The project was approved on August 21, 2014 by the Gaffrée and Guinle University Hospital Research Ethics Committee, under the Presentation Certificate for Ethical Appreciation 34119014.3.0000.5258, Judgement No. 759826, according to Resolution No. 466/12 of the Brazillian National Council of Health. Patients and volunteers who participated spontaneously read and signed an informed consent after receiving a detailed explanation of the study. There were no participants under the age of 18 or patients with mental illness, so it was not necessary to obtain informed consent from parents, guardians or legally authorized representatives. The study was conducted according to the guidelines of the Declaration of Helsinki.

## Results

From September 2014 and October 2017, 224 patients with cirrhosis and 53 healthy individuals were evaluated.

**Figure 1:**
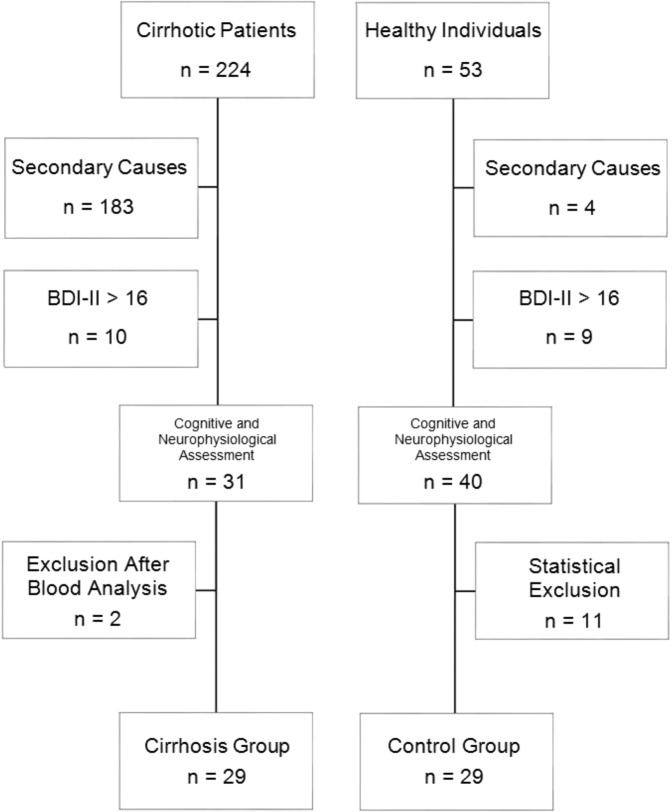
Selection of cirrhotic patients and controls.

**Figure 2:**
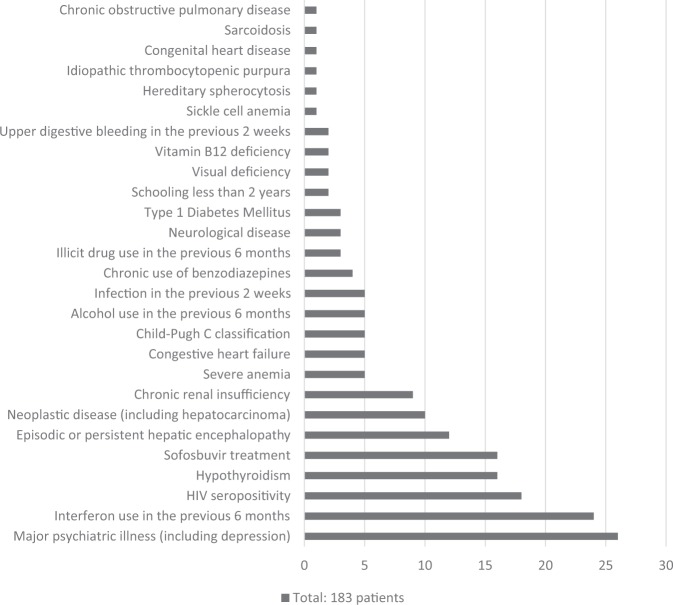
Secondary causes responsible for the exclusion of cirrhotics at first evaluation.

Among the cases, 183 patients were excluded in the first evaluation and 10 were excluded in the second evaluation, in a total of 193 cirrhotics (Fig. 1). This represented 82.5% of the sample. The reasons why each one was excluded in the first evaluation are described in Fig. 2. All cases excluded after the second evaluation were due to a BDI-II score greater than 16. Thus, 31 patients underwent cognitive and neurophysiological evaluation. Subsequently, blood was collected to determine TSH, free T4, cortisol, folic acid, vitamin B12, VDRL, and HIV status levels. Two cirrhotics demonstrated blood vitamin B12 levels below 250 pg/ml (161 pg/ml and 231 pg/ml) and were therefore excluded, leaving 29 cases in the final sample. In these, the minimum value of hemoglobin was 11.6 g/dl (hematocrit of 33.3%) and the average value of hemoglobin was 13.7 ± 1.6 g/dl (hematocrit of 40 ± 4.9%), which allowed to rule out the possibility that a severe anemia could be responsible for a cognitive or neurophysiological dysfunction.

Among healthy individuals, 4 were excluded in the first assessment because they had major psychiatric illness (depression) or history of use of psychotropic medication (benzodiazepines). Nine were excluded in the second assessment because they scored more than 16 in the BDI-II. Eleven were excluded for being too young (by at least 3 years), or for having a higher educational level (1 year or more) than the cirrhotic patients. Thus, a final sample of 29 people without cirrhosis participated as the control group (Fig. 1).

Within the patient group, the principal etiological causes identified were chronic hepatitis C (82.7%), chronic hepatitis B (6.8%), autoimmune hepatitis (6.8%) and cryptogenic disease (3.4%). The final sample had no cases of alcoholic cirrhosis, either alone or associated with other etiologies (Table 1).

**Table 1:** Etiology and Child-Pugh classification of patients with cirrhosis.

Etiology and Child-Pugh Classification	Frequency	
	Absolute (n)	Relative (%)
**Etiology**		
Alcoholic	0	0%
Cryptogenic	1	3.4%
Autoimmune hepatitis	2	6.8%
Hepatitis B	2	6.8%
Hepatitis C	24	82.7%
Hepatitis B and alcoholic	0	0%
Hepatitis C and alcoholic	0	0%
**Child-Pugh**		
5	24	82.7%
6	2	6.8%
7	3	10.3%

The average age of the patients with cirrhosis was 60 ± 9.5 years, which was identical to the final group of healthy individuals. The average educational level for cirrhotics and controls was 9.5 ± 3.3 years and 9.5 ± 3.0 years, respectively. The Mann-Whitney U test found no statistically significant difference between the groups with regard to age (*p* = 0.83) or educational level (*p* = 0.87).

The majority of the cohort (82.7%) consisted of patients with A5 Child-Pugh classification (Table 1). With respect to gender, 52% of the patients with cirrhosis were male and 48% were female, while 34% of the controls were male and 66% were female. The chi-square test did not verify a statistically significant difference between the cirrhosis patients and controls based on proportion of gender (*p* = 0.70).

Regarding the distribution of professional categories, the blue collar professionals corresponded to 41% of cirrhotics and 38% of controls. The white collar professions had a prevalence of 59% and 62%, respectively. The chi-square test showed no statistically significant difference between the cirrhosis patients and controls based on proportion of professional category (*p* = 0.71).

Concerning the BDI-II score, individuals with less than 12 points (suggestive of absent or minimal depression) constituted 86% of cases and 69% of controls. Participants with scores between 12 and 16 (suggestive of mild depression) constituted 14% and 31%, respectively. The chi-square test did not verify a statistically significant difference between the cirrhosis patients and controls based on proportion of mild depression (*p* = 1.00). Also, the distribution of compensated type 2 diabetes mellitus was 10% in both groups. The chi-square test showed no statistically significant difference (*p* = 0.28).

**Figure 3:**
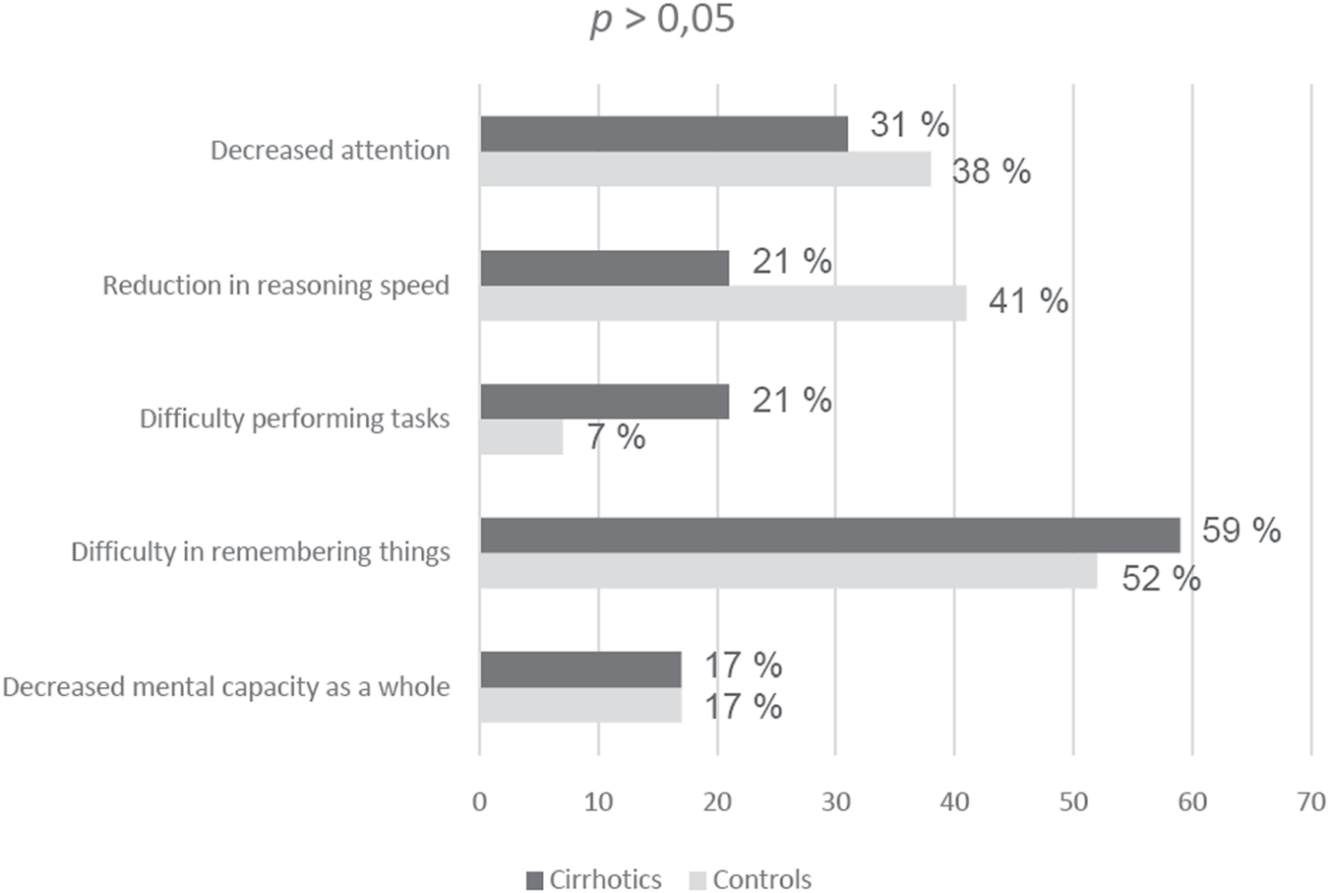
Proportion of cognitive complaints reported by cirrhotics and controls.

The prevalence of cognitive complaints reported by cirrhotic patients versus controls was evaluated using the chi-square test (Fig. 3), and no statistically significant difference in the symptoms reported by the two groups was detected (*p* > 0.05). Regarding spontaneous complaints, 3 patients reported asthenia, 1 complained of anxiety, 1 reported frequent drowsiness and 1 complained of sporadic headache.

Overall, there was no difference in the prevalence of cognitive changes detected by psychometric tests between the groups. Regarding the evaluation of global cognitive performance, patients with cirrhosis had a mean score of 27.5 ± 2.9 points (median of 28 points) on the MMSE, while the control group had a mean of 27.9 ± 2 points (median of 28 points). The Mann-Whitney U test showed no statistically significant difference between the two groups (*p* = 0.88) (Table 2). For MMSE items whose results had more than two categorical variables, the Mann-Whitney U test showed no statistically significant difference in the performance of both groups (*p* > 0.05) (Table 2). For MMSE items whose results had only two categorical variables, Fisher’s exact test only showed a statistically significant difference in “copying two intersected pentagons”, with the controls performing worse than cirrhotics (*p* = 0.02), a data that has no applicability in clinical practice (Table 3).

**Table 2:** Comparison of cognitive scores with more than two categorical variables between cases and controls.

Cognitive Test	Cirrhosis (n = 29)			Controls (n = 29)			*p*-value (Mann-Whitney U)
	Mean	SD^*^	Median	Mean	SD^*^	Median	
MMSE^**^							
Temporal orientation	4.9	0.3	5.0	4.9	0.3	5.0	0.65
Spatial orientation	5.0	0.2	5.0	5.0	0.2	5.0	1.00
Registration of three words	3.0	0.2	3.0	3.0	0.0	3.0	0.33
Subtraction of serial sevens	3.7	1.6	4.0	3.8	1.3	4.0	0.95
Word recall	2.3	0.9	3.0	2.7	0.5	3.0	0.11
Three step command	2.9	0.3	3.0	2.9	0.4	3.0	0.33
Total	27.5	2.5	28.0	27.9	2.0	28.0	0.88
**RAVLT**							
Learning (sum of A1 to A5)	39.0	8.6	40.0	43.5	7.6	45.0	0.09
Retroactive interference (A6/A5)	0.8	0.2	0.8	0.7	0.2	0.8	0.80
Proactive interference (B/A1)	0.9	0.3	0.8	1.0	0.3	1.0	0.15
Forgetting speed (A7/A6)	1.0	0.4	1.0	1.0	0.2	1.0	0.97
Recognition	9.6	2.9	10.0	10.3	2.3	10.0	0.37
Total	56.8	12.7	59.0	64.5	13.1	63.0	0.08
**PHES**							
DST	41.6	21.3	45.0	47.7	20.0	47.0	0.20
LTT (seconds)	129.7	57.0	123.0	122.3	43.2	122.0	0.87
LTT (errors)	60.5	29.2	52.0	63.1	34.1	58.0	0.84
LTT (German and Chinese versions)^***^	75.9	47.9	60.6	76.9	54.2	61.6	0.84
LTT (Italian version)^***^	7458.0	4758.4	5934.0	7571.0	5399.7	6072.0	0.85
LTT (Spanish and Portuguese versions)^***^	190.1	57.5	174.0	185.4	52.2	172.0	0.91
SDT	77.0	24.8	74.0	76.5	31.8	68.0	0.74
TMT-A (seconds)	67.7	33.9	55.1	54.9	31.8	49.1	0.09
TMT-B (seconds)	161.4	99.5	99.0	178.9	96.4	180.0	0.71
Total (German and Chinese versions)	+0.7	±2.9	0.0	+0.8	±2.7	+0.2	0.67
Total (Italian version)	+0.7	±2.9	−0.1	+0.8	±2.7	+0.2	0.67
Total (Spanish and Portuguese versions)	+0.8	±3.1	−0.1	+0.8	±2.6	+0.5	0.59

^*^Standard deviation.

^**^All cases and controls scored 2 points in the item “naming two objets” on MMSE.

^***^Z score.

**Table 3:** Comparison of cognitive scores with two categorical variables between cases and controls.

Cognitive Test	Cirrhosis (n = 29)		Controls (n = 29)		p-value (Fisher’s exact test)
	n	%	n	%	
MMSE*					
**Repetition**					
0 points	0	0	1	3.5	0.33
1 point	29	100	28	96.5	
**Writing a sentence**					
0 points	3	10	2	7.0	1.00
1 point	26	90	27	93.0	
**Copying two intersected pentagons**					
0 points	5	17	9	31.0	**0.02**
1 point	24	83	20	69.0	

*All cases and controls scored 1 point in the item “reading a sentence” on MMSE.

Concerning the performance on RAVLT, the Mann-Whitney U test showed no statistically significant difference between cases and controls in “learning”, “retroactive interference”, “proactive interference”, “forgetting speed” and “recognition” items (*p* > 0.05). Cirrhotics had an average score of 56.8 ± 12.7 points (median of 59 points), while the control group had an average score of 64.5 ± 13.1 points (median of 63 points), with no relevant statistical difference (*p* = 0.08) (Table 2).

The final score of PHES showed a mean of +0.7 ± 2.9 (German and Chinese methodology), +0.7 ± 2.9 (Italian methodology) and +0.8 ± 3.1 (Spanish and Portuguese methodology) for cirrhotic patients, while the control group presented a mean of +0.8 ± 2.7 (German and Chinese methodology), +0.8 ± 2.7 (Italian methodology) and + 0.8 ± 2.6 (Spanish and Portuguese methodology). The Mann-Whitney U test showed no statistically significant difference between the two groups using the methodologies of those countries (*p* > 0.05). Regarding the LTT item, there was no difference between cases and controls in time, number of errors and Z scores calculated according to the German, Chinese, Italian, Portuguese and Spanish versions (*p* > 0.05). There was also no statistically significant difference between the groups in the DST, SDT, TMT-A and TMT-B items (*p* > 0.05) (Table 2).

The electroencephalogram of all participants showed a reactive posterior alpha rhythm, without inversion of the frequency-amplitude gradient. TMBEA demonstrated, in both cases and controls, dominant alpha/beta 1 frequency, with an average of 12.9 ± 0.5 Hz (median of 12.9 Hz). Mann-Whitney U test showed no statistically significant difference between the groups (*p* = 0.76) (Table 4). Concerning the delta, theta, alpha, beta 1, beta 2 and beta 3 relative frequencies, there was no statistically significant difference between cases and controls (*p* > 0.05) (Table 5). Regarding the absolute amplitudes of the frequency bands, the Mann-Whitney U test only showed a statistically significant difference in the beta 2 frequency, with cirrhotics presenting a mean of 2.2 ± 0.8 µV (median 1.9 µV), slightly lower than the control group, which had a mean of 2.5 ± 0.8 µV (median 2.3 µV) (*p* = 0.02) (Table 6). Concerning the relative amplitudes of the frequency bands, there was no statistically significant difference between cases and controls (*p* > 0.05) (Table 7). When analyzing the theta/beta ratio, the Mann-Whitney U test showed no statistically significant difference between groups for the results obtained with beta 1, beta 2 and beta 3 frequencies (*p* > 0.05) (Table 8).

**Table 4:** Mean dominant frequency during TMBEA in cirrhotics and controls.

Mean Dominant Frequency (Hz)	Group		*p*-value (Mann-Whitney U)
	Cirrhosis (n = 29)	Controls (n = 29)	
Mean	12.9	12.9	0.76
Standard deviation	0.5	0.5	
Minimal	12.1	12.1	
Median	12.9	12.9	
Maximum	14.3	14.3

**Table 5:** Relative frequencies during TMBEA in cirrhotics and controls.

Relative Frequency (%)	Cirrhosis (n = 29)			Controls (n = 29)			*p*-value (Mann-Whitney U)
	Mean	SD*	Median	Mean	SD*	Median	
Delta (less than 4 Hz)	2.1	0.7	2.0	1.8	0.3	1.6	0.07
Theta (4 to 7 Hz)	5.6	1.9	4.0	5.6	1.8	4.0	1.00
Alpha (8 to 12 Hz)	9.1	1.0	9.0	9.6	1.0	9.8	0.13
Beta 1 (13 to 16 Hz)	14.7	2.9	12.6	14.1	2.7	12.6	0.22
Beta 2 (17 to 20 Hz)	20.1	0.3	20.0	20.0	0.0	20.0	0.33
Beta 3 (21 to 28 Hz)	26.3	0.3	26.2	26.3	0.5	26.2	0.28

*Standard deviation.

**Table 6:** Absolute amplitudes during TMBEA in cirrhotics and controls.

Absolute Amplitude (µV)	Cirrhosis (n = 29)			Controls (n = 29)			*p*-value (Mann-Whitney U)
	Mean	SD*	Median	Mean	SD*	Median	
Delta (less than 4 Hz)	7.4	4.9	5.4	9.4	7.6	5.7	0.32
Theta (4 to 7 Hz)	6.3	3.2	5.6	6.8	3.9	4.7	0.76
Alpha (8 to 12 Hz)	6.5	2.6	5.7	7.3	3.1	6.5	0.20
Beta 1 (13 to 16 Hz)	3.4	1.2	3.1	3.8	1.2	3.7	0.06
Beta 2 (17 to 20 Hz)	2.2	0.8	1.9	2.5	0.8	2.3	**0.02**
Beta 3 (21 to 28 Hz)	1.5	0.5	1.4	1.6	0.5	1.4	0.19

*Standard deviation.

**Table 7:** Relative amplitudes during TMBEA in cirrhotics and controls.

Relative Amplitude (%)	Cirrhosis (n = 29)			Controls (n = 29)			*p*-value (Mann-Whitney U)
	Mean	SD*	Median	Mean	SD*	Median	
Delta (less than 4 Hz)	21.3	8.3	17.5	22.1	8.6	18.9	0.59
Theta (4 to 7 Hz)	20.1	5.1	19.0	19.0	3.4	18.7	0.60
Alpha (8 to 12 Hz)	25.4	7.2	26.0	25.2	6.1	25.1	0.92
Beta 1 (13 to 16 Hz)	20.3	3.3	20.2	20.5	4.1	20.2	0.84
Beta 2 (17 to 20 Hz)	11.6	3.0	11.2	11.9	3.2	11.8	0.68
Beta 3 (21 to 28 Hz)	8.2	2.3	8.2	7.9	2.3	8.0	0.70

*Standard deviation.

**Table 8:** Theta/beta ratio during TMBEA in cirrhotics and controls.

Ratio	Cirrhosis (n = 29)			Controls (n = 29)			*p*-value (Mann-Whitney U)
	Mean	SD*	Median	Mean	SD*	Median	
Theta/Beta 1	1.1	0.5	1.0	1.0	0.3	1.0	0.53
Theta/Beta 2	2.1	1.1	1.8	2.0	0.8	1.8	0.91
Theta/Beta 3	3.3	1.9	2.9	3.1	1.3	2.8	0.98

*Standard deviation.

**Figure 4:**
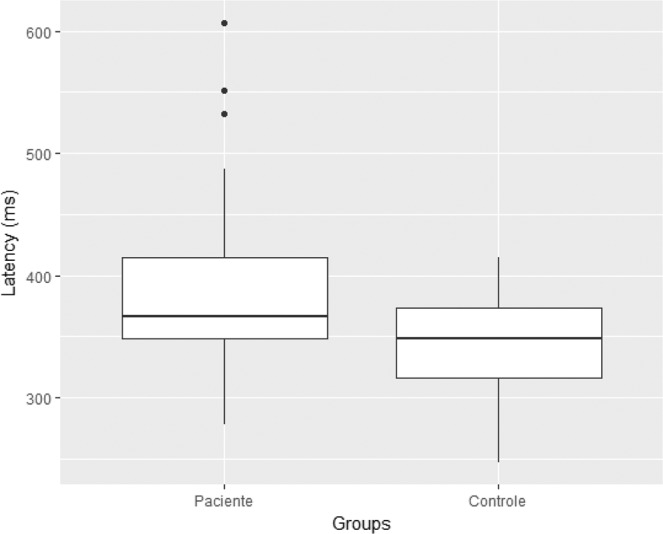
Z scores* for P3b latency in cirrhotics and controls. ${}^{\ast }Z\,score=\{[value\,found-(250+1.4\times age)]/40\}$.

The analysis of the gross values for latency and amplitude of P3a and P3b showed only a statistically significant difference in the amplitude of the P3b component, and the cirrhotic group had an average of 8.5 ± 4.3 µV (median of 7.5 µV), while the control group had an average of 11.4 ± 5.7 µV (median of 8.5 µV) (*p* = 0.04) (Table 9). When the P3b latency was adjusted for age, following the norms recommended by the International Federation of Clinical Neurophysiology, the Z scores demonstrated not only a statistically significant difference in the Mann-Whitney U test, but also extremely significant levels (*p* < 0.01). Z scores of cirrhotic patients exhibited a mean of 385 ± 78 milliseconds (median of 366.6 ms), while controls had a mean of 346.2 ± 42.8 milliseconds (median of 348.2 ms) (Table 10 and Fig. 4).

**Table 9:** Gross values of P300 in cirrhotics and controls.

P300	Cirrhosis (n = 29)			Controls (n = 29)			*p*-value (Mann-Whitney U)
	Mean	SD*	Median	Mean	SD*	Median	
Latency of P3a (ms)	359.3	73.0	340.7	323.8	39.9	327.4	0.07
Latency of P3b (ms)	385.9	78.0	367.4	347.1	42.8	349.1	0.06
Amplitude of P3b (µV)	8.5	4.3	7.5	11.4	5.7	8.9	**0.04**

*Standard deviation.

**Table 10:** Z scores* for P3b latency in cirrhotics and controls.

Z score*	Group		*p*-value (Mann-Whitney U)
	Cirrhosis (n = 29)	Controls (n = 29)	
Mean	385.0	346.2	**<0.01**
Standard deviation	78.0	42.8	
Minimal	278.2	246.4	
Median	366.6	348.2	
Maximum	607.0	415.0	

*Z score = {[value found - (250 + 1.4 x age)] / 40}.

The Kruskal-Wallis test showed that cognitive assessment was influenced by gender, education, professional category, high scores on transient hepatic elastography, and scores suggestive of mild depression in BDI-II, while neurophysiological evaluation was influenced by age, gender, educational level, Child-Pugh classification and scores suggestive of mild depression in BDI-II. Of particular importance, when cirrhosis patients with and without scores suggestive of mild depression in BDI-II were compared, it was found that beta 2 relative frequency was increased in those who had scores between 12 and 16 points (*p* < 0.01). The etiology of cirrhosis, association with compensated type 2 diabetes mellitus and history of interferon use did not change patient’s performance. Despite the observed differences, the cases and controls were adequately paired according to age, gender, educational level, professional category, association with compensated type 2 diabetes mellitus and scores suggestive of mild depression in BDI-II to avoid confusion bias.

## Discussion

The primary objective of the present study was to compare the performance of MMSE, RAVLT, PHES (German, Chinese, Italian, Spanish and Portuguese versions), TMBEA and P300 in the detection of minimal hepatic encephalopathy in paired samples in Brazil. The secondary objectives were to describe the cognitive functions and neurophysiological parameters that were the most compromised in the initial phases of cirrhosis, to compare the performance of cirrhotic patients with the performance of healthy individuals (controls), and to identify the standardized tests that detected these changes.

In the initial sample, there was a high prevalence of comorbidities responsible for cognitive and neurophysiological deficits. As a result, 82.5% of cases were excluded. The most frequent causes were: major psychiatric illness, mainly depression (16%); interferon use in the last 6 months (10.7%); HIV seropositivity (8%); hypothyroidism (7.1%); and hepatic encephalopathy at any time during clinical evolution (5.3%) (Fig. 2). All patients with hepatitis C who were using sofosbuvir were excluded, since there is a current controversy about whether their effects may lead to cognitive improvement^27^. On the other hand, the exclusion of autoimmune hepatitis or chronic B hepatitis using prednisone, tenofovir or entecavir was only necessary in cases where psychiatric disorders or renal failure were present.

This high exclusion rate was much greater than that described by studies commonly found in literature. It should be noted that the prevalence of secondary causes of cognitive and neurophysiological deficits in those patients was similar to that found in a previous study conducted in Brazil, where 84% of the preselected cases were later excluded^4^. Therefore, it is highly recommended that cirrhotics make a minimum screening for these conditions.

Of the patients admitted after the initial evaluation, 10 cases (24.3%) were excluded because they had more than 16 points in BDI-II, i.e., scores suggestive of moderate or severe depression (Fig. 1). A significant number may have false-positive results, as a study with untreated hepatitis C patients showed that somatic symptoms such as “energy loss”, “appetite disorders” and “sleep disturbances” continue to be evaluated in BDI-II, and still have significant weight in the final score^28^. However, it was decided to use this instrument, since little emphasis is given, in clinical practice, to emotional and social adjustment conditions in the early stages of cirrhosis.

A previous Brazillian study of 138 untreated hepatitis C patients showed that, apart from the secondary causes, little or no cognitive dysfunction remained evident in the sample^29^. The most important criticism established in relation to this work was that the use of very restrictive criteria regarding the coexistence of depressive symptoms could mask initial deficits in the disease, since dopamine and serotonin metabolism disorders would constitute some of the earliest changes in the neurobiology of hepatic encephalopathy^30^. As a result, the present work opted for the establishment of less strict selection criteria, accepting participants with BDI-II scores between 12 and 16, i.e., suggestive of mild depression.

Evidently, the results would be strongly biased by the inclusion of patients with a BDI-II score between 12 and 16, if they were not appropriate paired with the control group. Despite there was no statistically significant difference between the groups, it is interesting to note that, in the present study, healthy participants had a higher percentage of scores suggestive of mild depression than cirrhotic patients (31% versus 14%, respectively).

The final sample consisted, in 82.7% of cases, of patients with A5 Child-Pugh classification, i.e., the initial stage of cirrhosis. The mean and minimum hemoglobin and hematocrit values allowed to rule out the presence of severe anemia, which could serve as a confounding factor for poor performance. Although not part of the exclusion criteria, the sample did not present any case of alcoholic cirrhosis, either alone or associated with other etiologies (Table 1). This is believed to be due to its strong association with major psychiatric illnesses, especially depression, which should lead, in clinical practice, to more careful screening of this condition through active investigation, with questionnaires such as BDI-II.

In addition to scores in BDI-II suggestive of mild depression, other factors that affected cognitive or neurophysiological performance were age, gender, educational level, professional category, Child-Pugh classification, and high scores on transient hepatic elastography. Again, appropriate matching with the control group regarding age, gender, educational level and professional category helped to minimize confusion bias.

The main symptoms reported by cirrhotics were “decreased attention”, “reduction in reasoning speed” and “decreased mental capacity as a whole”, although there was no statistically significant difference in the prevalence of cognitive complaints between cases and controls. Figure 3 shows that symptoms such as “decreased attention” and “reduction in reasoning speed” were more common in the control group. These data allow to conclude that, in the early stages of cirrhosis, spontaneous reporting is not a reliable parameter for the presence of minimal hepatic encephalopathy.

Comparing the groups, it appears that no significant psychometric test was altered at early stages of the disease (Tables 2 and 3). This contrasts with the results of a previous study at the same unit, which detected changes in the total score of MMSE, “learning”, “retroactive interference” and “recognition” items in RAVLT, TMT-A and TMT-B. Considering that, in both studies, the average age and the educational level were close, the higher percentage of patients with more advanced Child-Pugh classification seems to have been the main determining factor for this difference. In fact, the final study sample consisted predominantly of patients with A5 Child-Pugh classification (82.7%), with less patients with B6 (6.8%) and B7 (10.3%) Child-Pugh classification (Table 1). On the other hand, in the previous study the percentage of patients with A5 Child-Pugh classification was lower (69%), with a higher prevalence of cirrhotics with A6 (20.7%), B7 (17.2%) and even B8 (3.4%) Child-Pugh classification^4^. These data led the current work to compare the cirrhotic subgroups. It was shown that a higher Child-Pugh classification had negative influence in the neurophysiological assessment and high scores on transient hepatic elastography had negative influence in the cognitive performance, which provides indirect evidence that the composition with a more advanced Child-Pugh classification may justify the discrepancy observed between the studies.

It was curious that, in MMSE, the control group performed significantly worse than the cirrhosis patients in “copying two intersected pentagons” (Table 3). In fact, there is a reasonably plausible explanation for this finding: the control group had a higher percentage of female participants than the cirrhotic group (66% versus 48%, respectively). Although no statistical difference in proportion of gender was found between cases and controls, it is known that females perform worse than males in tasks that require visuospatial skills^31^, a confusion bias that could justify the poorer performance of the control group.

Overall, cirrhotics performed very similarly to controls on the remaining items of MMSE (Tables 2 and 3). These findings are consistent with those reported by a Polish study^12^, which evaluated 101 cirrhosis patients, and disagreeing with the results of an Austrian study^11^, which found a low MMSE performance in 22 patients with B and C Child-Pugh classification who underwent portosystemic shunt.

Verbal episodic memory was specifically assessed by RAVLT. Long-term potentiation depends mainly on the proper functioning of the glutamatergic system in the perforating pathway and of the cholinergic system projecting from the basal forebrain to the medial portion of the temporal lobe^32^. It is known that, in cirrhosis, glutamatergic hyperactivity can induce neuronal death^7,33^. In addition, there is also a decrease in acetylcholine levels, which enhances the effects of increased GABAergic tonus^34^. Therefore, the applicability of RAVLT as a screening instrument for a medial temporal system dysfunction in this disease was investigated.

Although cirrhotics showed poorer learning performance and total RAVLT score, the difference was not statistically significant (Table 2). Again, these findings contrast with those of a previous study at the same unit, whose sample had a higher proportion of cases with more advanced Child-Pugh classification, which found “learning”, “retroactive interference” and “recognition” deficits^4^. A Spanish group also described a statistically significant difference in the total RAVLT score in 97 patients with advanced cirrhosis and 23 patients undergoing liver transplantation^35^, which indicates that the stage of the disease could be a key performance variable.

Regarding PHES, the application of the German, Chinese, Italian, Portuguese and Spanish versions in this sample showed that the scores do not allow an adequate identification of patients with minimal hepatic encephalopathy in the early stages of the disease. Overall, patients with cirrhosis performed worse on its five items, which suggests a possible decrease in psychomotor processing speed, but the difference was not statistically significant.

Contrary to what was described in the German PHES validation^16^, the number of LTT errors did not show greater accuracy than LTT execution time in cirrhotic patients (Table 2). Thus, the present study tends to agree with the arguments of an Italian group^18^, who argue that the time of execution and the number of errors in LTT are intrinsically related. Indeed, there were no cases in the present study that exhibited an isolated reduction of dexterity in fine hand movements caused by mini-asterixis. Such observations, however, need corroboration from studies with larger cohorts.

Concerning TMT-A and TMT-B, the results of previous work had shown that both tests have prolonged execution time in more advanced cirrhosis, with significant difference in relation to controls^4^. In the present sample, it was not possible to prove statistical difference between the groups, which could be explained by a lower prevalence of advanced stages of the disease. A major obstacle in the interpretation of TMT-A and TMT-B is the strong influence of the educational level, which was corroborated by the present study. These data are in agreement with those reported by a group from São Paulo, which studied the applicability of TMT-A and TMT-B in 48 patients with cirrhosis, and found that, in populations with low educational level, there is an increase in the time to perform the tests^36^, and are also in line with a standardization project conducted on 313 healthy volunteers in Rio de Janeiro, where age and schooling were critical to their performance^37^.

Regarding TMBEA, both cases and controls had a dominant mean alpha/beta 1 frequency, with an average of 12.9 ± 0.5 Hz, which indicates that the occipito-parietal cortical centers, influenced by the thalamic pacemaker neurons projections, remain normal in the early stages of cirrhosis (Table 4). No statistically significant difference was found in the percentage of relative frequencies between the groups (Table 5). There is, however, the possibility that a higher proportion of controls with scores suggestive of mild depression in BDI-II (31%) may have led to increased beta frequency in this group: when cirrhosis patients with and without scores suggestive of mild depression in BDI-II were compared, it was found that beta 2 relative amplitude was increased precisely in those who had scores between 12 and 16. The same beta 2 frequency presented its relative amplitude significantly high in the control group (Table 6). Since the beta frequency is associated with an increase in GABAergic tonus^38,39^, and a reduction in serotoninergic and dopaminergic activity potentializes it^32^, it is possible that depression may have acted as a confusion bias, despite adequate pairing.

The theta frequency, which reflects modulation of cortical activity by cholinergic pathways originating from the basal forebrain and ascending reticular activating system during states of cortical depression^40^, had no change in its relative frequency or amplitude in cirrhosis (Tables 5 and 7). It was expected that a reduction in cholinergic activity and an increase in GABAergic tonus in cirrhosis would cause, respectively, decreased hippocampal theta activity and increased beta activity. Therefore, the theta/beta ratio could be decreased. However, the comparison of this ratio between the groups showed no statistically significant difference (Table 8).

The most important data of the present study concerns the comparison of P300 latencies between cases and controls. During the analysis of its gross values, it was already observed that patients with cirrhosis had higher latencies for P3a and P3b components, although the difference was not statistically significant (Table 9). As age may cause a physiological increase in P300 latencies^26^, the values obtained from the P3b component (reference for P300 latency) were corrected for it, according to the equation Z score = {[value found - (250 + 1.4 x age)] / 40}, which is recommended by the International Federation of Clinical Neurophysiology^25^. There was then a marked difference in P3b component latency between the groups (Fig. 4), with cirrhotics showing a mean of 385 ± 78 milliseconds (median of 366.6 ms), and controls showing a mean of 346.2 ± 42.8 milliseconds (median of 348.2 ms), with a level of statistical significance less than 0.01 (Table 10). These data are in agreement with those described by São Paulo, Polish and Indian groups, who compared the performance in TMT-A, TMT-B, RAVLT and P300, verifying superiority of the latter;^10,36,41^ and contrasts with the results of an Italian group, who did not verify P300 superiority over SDT, TMT-A, TMT-B and TMBEA^9^.

The amplitude of the P3b component also showed a statistically significant difference between the groups, with cirrhosis patients showing a mean of 8.5 ± 4.3 µV (median of 7.5 µV), while the controls showed a mean of 11.4 ± 5.7 µV (median 8.9 µV) (Table 9). However, two important criticisms should be made regarding this finding: the normal range of P300 amplitude may vary widely in literature, extending between limits as extreme as 5 to 20 µV, and some commercially available software calculate the amplitude of P300 from the midline of the plot, although it should be from the lowest point of the N200 (N2) curve, as preconized by the International Federation of Clinical Neurophysiology^25^. For these reasons, despite the statistically significant difference found in the present study, it is not prudent to use P300 amplitude alone as a diagnostic parameter for minimal hepatic encephalopathy.

From a neurophysiological perspective, the latency of P3a component (generated by frontal attentional circuits during information processing) would constitute a kind of passive comparison system for auditory stimuli, while latency of P3b component (an indirect measure of cortical activity in supramarginal gyrus and, possibly, hippocampal circuits), would be responsible for coding new memory traces and recognition^42^. Therefore, a comparison was made between learning and recognition in RAVLT with age-adjusted P300 latency, as both assess medial temporal system activity.

In the present sample (predominantly composed of patients with A5 Child-Pugh classification), the age-adjusted P3b latency demonstrated expressive superiority over RAVLT, which was expected, considering that cognitive changes are the emerging result of neurophysiological dysfunctions. The data suggest that dysfunctions governed by the glutamatergic system and maybe cholinergic system in the medial temporal system^32^, may represent some of the earliest changes in minimal hepatic encephalopathy, a fact also observed clinically in a previous study with more advanced stages of the disease^4^.

Moreover, the adoption of very restrictive selection criteria in the study could be responsible for the fact that many patients in the final sample were particularly at low risk for minimal hepatic encephalopathy. Obtaining a sample where it was extremely difficult to define which patients had minimal hepatic encephalopathy or not, even using psychometric and neurophysiological tests capable of detecting subtle changes, may help to clarify which alterations could serve as earlier biomarkers for this disorder. This is, however, an observational cross-sectional study and longitudinal studies with larger groups are needed to confirm these suspicions.

In addition to the small number of cases, due to the rigidity of the exclusion criteria, other drawbacks can be pointed out: the fact that the control group did not undergo a blood sample analysis as the cirrhosis group; the fact that the study was not conducted in a blinded way; and, mainly, despite the fact that registering P300 is non-invasive and requires a maximum of 20 minutes, the facilities to record it are uncommon in departments of liver diseases, needing an adequate integration with a Clinical Neurophysiology service. Nonetheless, minimal hepatic encephalopathy is a transdisciplinary disease, and the authors encourage greater exchanges between different scientific areas to better understand its pathophysiology.

On the other hand, the research has the following positive points: cirrhosis patients were paired to healthy controls on critical variables such as age, education, gender, professional category, scores in BDI-II suggestive of mild depression, association with compensated type 2 diabetes mellitus, and sociodemographic characteristics, which could influence the performance in cognitive and neurophysiological tests; non-invasive instruments were used, with low cost for the Brazilian population; and the cognitive and neurophysiological battery was larger and more diverse than is normally reported in literature, which allowed the evaluation of potential effects of minimal hepatic encephalopathy on a variety of domains. Comparison with universal instruments for different cultures in the initial stages of cirrhosis allows a better insight into the neural networks compromised earlier in minimal hepatic encephalopathy. It can be argued that pencil-and-paper tests are old-fashioned, but they are public (therefore more available to different countries) and their accuracy is equal to that of computerized tests (which traditionally require the payment of a software license and have fewer validation studies).

In conclusion, in patients with early-stage cirrhosis, age-adjusted P300 latency was shown to be superior to neuropsychological assessment with MMSE, RAVLT, and PHES according to German, Chinese, Italian, Spanish, and Portuguese versions. Age-adjusted P300 latency was also superior to neurophysiological evaluation by TMBEA. Comparison with controls paired on critical variables that could interfere with the assessment has shown that, in cirrhosis, there is also a decrease in P300 amplitude. However, this variable is more difficult to obtain and interpret in clinical practice. Taken together, these findings suggest that a dysfunction in the medial temporal system may be an important feature of minimal hepatic encephalopathy, and this requires further study.

## Data availability

The datasets generated during and/or analysed during the current study are available from the corresponding author on reasonable request.
